# Supplementary material for: Different viral effectors suppress hormone-mediated antiviral immunity of rice coordinated by OsNPR1
Source: Nat Commun. 2023 May 25;14:3011. doi: 10.1038/s41467-023-38805-x (PMC10213043; doi:10.1038/s41467-023-38805-x)
Supplement: Supplementary file 1 — Supplementary Information [file 41467_2023_38805_MOESM1_ESM.pdf]

Supplementary Information for

Different viral effectors suppress the hormone-mediated antiviral immunity of rice coordinated by OsNPR1

Hehong Zhang[1], Fengmin Wang[1], Weiqi Song[1], Zihang Yang[1], Lulu Li[1], Qiang Ma[1], Xiaoxiang Tan[1], Zhongyan Wei[1], Yanjun Li[1], Junmin Li[1], Fei Yan[1], Jianping Chen[1#] and Zongtao Sun[1#]

[1]State Key Laboratory for Managing Biotic and Chemical Threats to the Quality and Safety of Agro-products, Key Laboratory of Biotechnology in Plant Protection of MARA and Zhejiang Province, Institute of Plant Virology, Ningbo University, Ningbo 315211, China

[#] Authors for correspondence:

Zongtao Sun, sunzongtao@nbu.edu.cn;

Jianping Chen, jianpingchen@nbu.edu.cn

Supplementary Figures: 17

Supplementary Table: 2

Supplementary Figures 1- 17

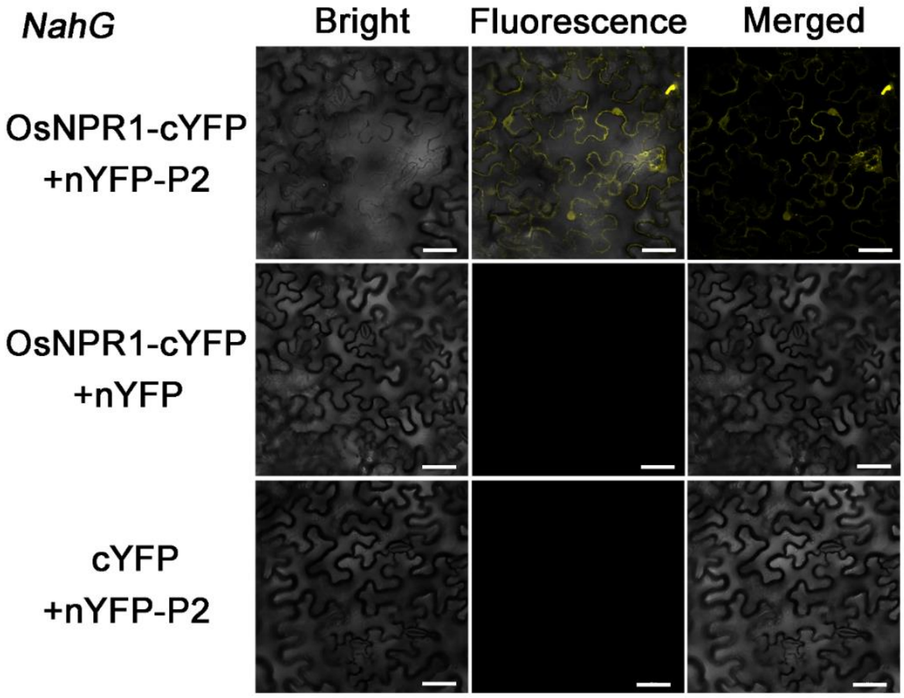

Supplementary Fig. 1. P2 interacts with OsNPR1 in a SA-independent manner. BiFC assays confirming the interactions of OsNPR1 with RSV P2 protein in *NahG N. benthamiana* leaves. The images were captured by confocal microscope at 48 h post inoculation. Scale bar = 50 µm. Experiments were repeated three times with the similar results.

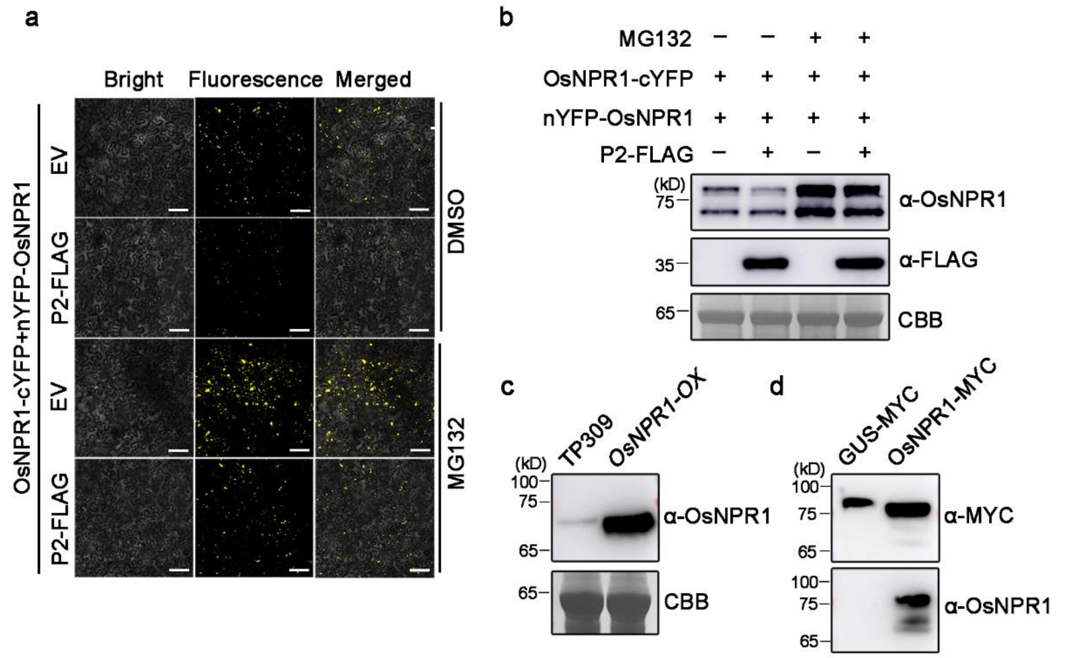

Supplementary Fig. 2. P2 influences the protein stability of OsNPR1. a. BiFC assays showing that P2 protein influences the formation of OsNPR1 oligomers in *N. benthamiana* leaves. OsNPR1-cYFP and nYFP-OsNPR1 were co-expressed with or without P2-FLAG in tobacco leaves. The leaves were injected with 50 μM MG132, and DMSO as control at 24 hpi. The images were captured by confocal microscopy at 48 hpi. Scale bar = 50 μm. **b.** The relative accumulation levels of OsNPR1 proteins co-expressed with or without P2 protein in the leaves of *N. benthamiana* analyzed by immunoblot using anti-OsNPR1 antibody. CBB staining was used as a loading control to monitor input protein amounts. **c, d.** The specificity of OsNPR1 antibody using western blotting assays in rice and *N. benthamiana* leaves. Endogenous OsNPR1 protein levels in WT (TP309) and *OsNPR1-OX* rice plants (c). Total proteins were extracted and then immunoblotted by gel blot with anti-OsNPR1 antibody (d). OsNPR1-MYC or GUS-MYC was expressed in *N. benthamiana* by agroinfiltration, respectively, and then the extracts were obtained for western blotting at 48 hpi, with anti-MYC and anti-OsNPR1 antibodies. Experiments in **a-d** were repeated three times with the similar results. Source data including uncropped scans of gels (**b-d**) are provided in the Source data file.

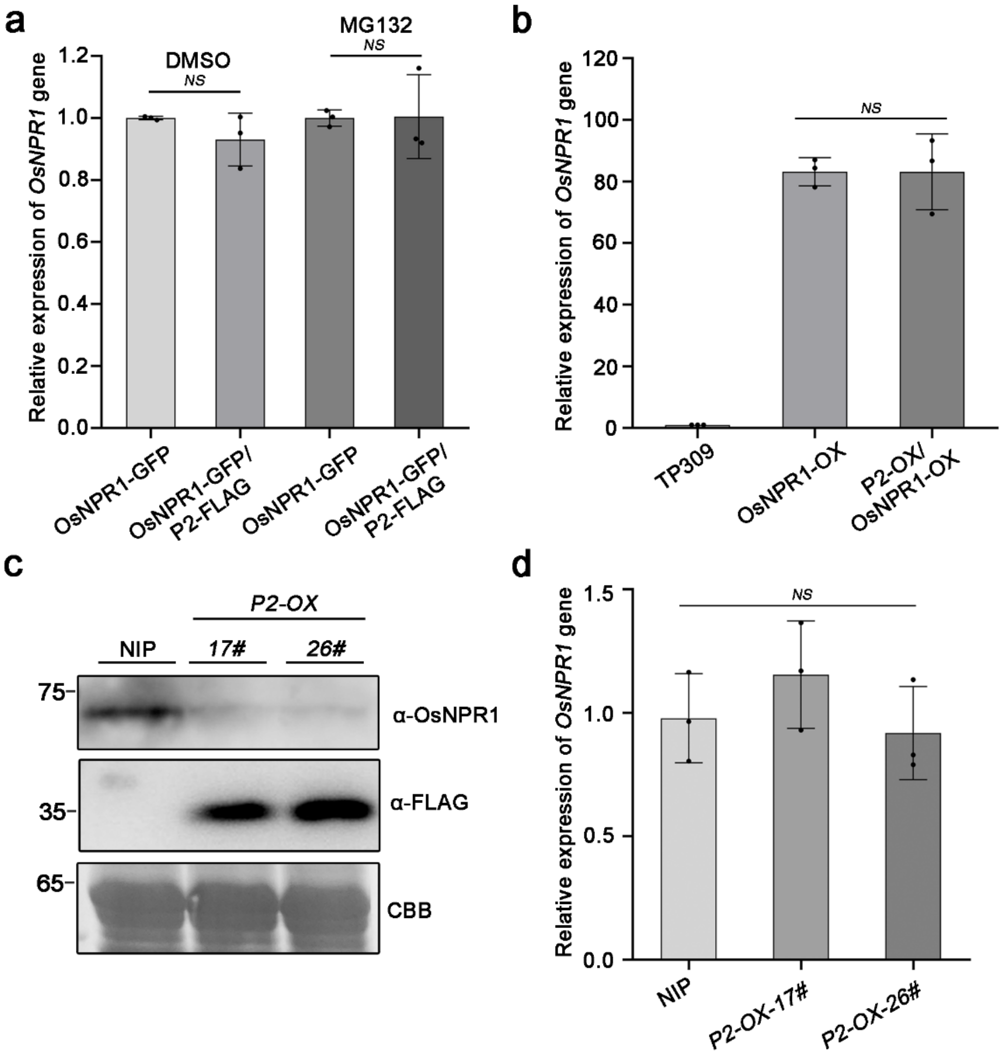

Supplementary Fig. 3. The relative expression of *OsNPR1* gene. a. The *OsNPR1* transcript levels with or without P2-FLAG as detected by RT-qPCR. Error bars represent SD, values are means ± SD (n = 3 biologically independent replicates per genotype). Significant differences were analyzed using one-way ANOVA followed by Tukey's multiple comparisons test. * at the columns indicate significant differences ($p \leq 0.05$). *NS*, no significance. **b.** The relative expression levels of *OsNPR1* gene in *OsNPR1-OX, OsNPR1-OX/P2-OX, P2-OX* transgenic plants and WT rice plants. Error bars represent SD, values are means ± SD (n = 3 biologically independent replicates per genotype). Significant differences were analyzed using one-way ANOVA followed by Tukey's multiple comparisons test. * at the columns indicate significant differences ($p \leq 0.05$). *NS*, no significance. **c.** The protein

levels of OsNPR1 in *P2-OX* transgenic and NIP rice plants. **d.** The transcription levels of *OsNPR1* in *P2-OX* transgenic and NIP rice plants. Error bars represent SD, values are means ± SD (n = 3 biologically independent replicates per genotype). Significant differences were analyzed using one-way ANOVA followed by Tukey's multiple comparisons test. * at the columns indicate significant differences ($p \leq 0.05$). *NS*, no significance. Experiments in **c** were repeated three times with the similar results. Source data including uncropped scans of gels **a** and *p* values of statistic tests (**a, b** and **d**) are provided in the Source data file.

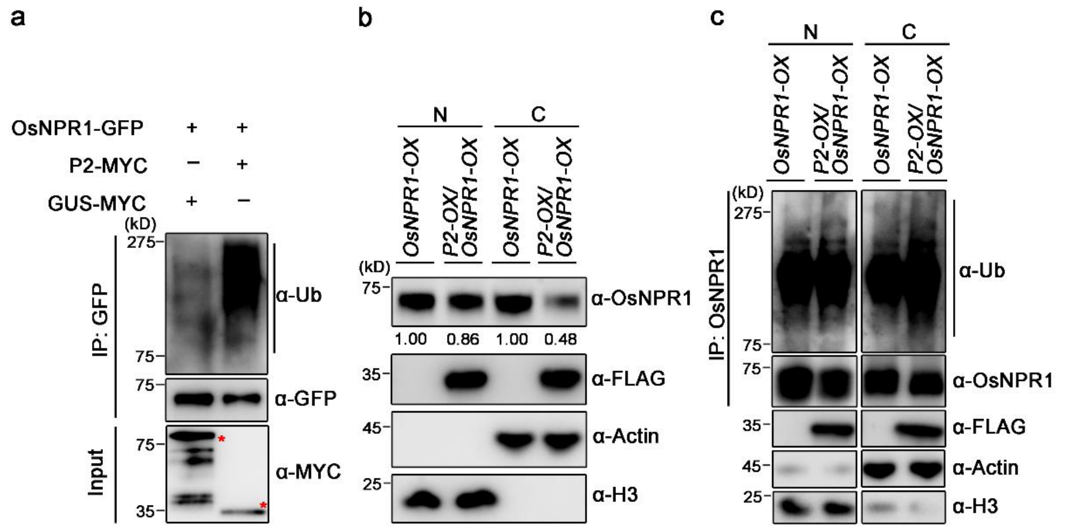

Supplementary Fig. 4. P2 promotes OsNPR1 poly-ubiquitination in *N. benthamiana*. a. OsNPR1-GFP was co-expressed with or without P2-FLAG in *N. benthamiana* by agroinfiltration and then treated with 100 μM MG132 for 5 h. The protein extracts were precipitated with GFP beads. The similar amounts of GFP precipitated by the beads were separated by SDS-PAGE gel and analyzed by immunoblotting using anti-Ubiquitin (Ub), anti-GFP and anti-MYC antibodies. Bands shown in figure are indicated by red asterisk. **b.** Nuclear-cytoplasmic fractionation analysis of OsNPR1 accumulation in *OsNPR1-OX* and *OsNPR1-OX/P2-OX* plants. **c.** Nuclear-cytoplasmic fractionation analysis of the influence of P2 on OsNPR1 ubiquitination. The protein extracts of *OsNPR1-OX/P2-OX* and *OsNPR1-OX* rice leaves were extracted in buffer containing 100 μM MG132 and 10 mM DTT and precipitated with Protein A/G OsNPR1 antibody beads. Similar amounts of OsNPR1 precipitated by the antibody beads were separated by SDS-PAGE gel and analyzed by immunoblotting using anti-Ubiquitin (Ub), anti-OsNPR1, anti-FLAG, anti-Actin and anti-H3 antibodies. Actin was used as a cytoplasmic marker, and histone H3 was used as a nuclear marker. N, nuclear fraction; C, cytoplasmic fraction. Experiments in **a-c** were repeated three times with the similar results. Source data including uncropped scans of gels **a-c** are provided in the Source data file.

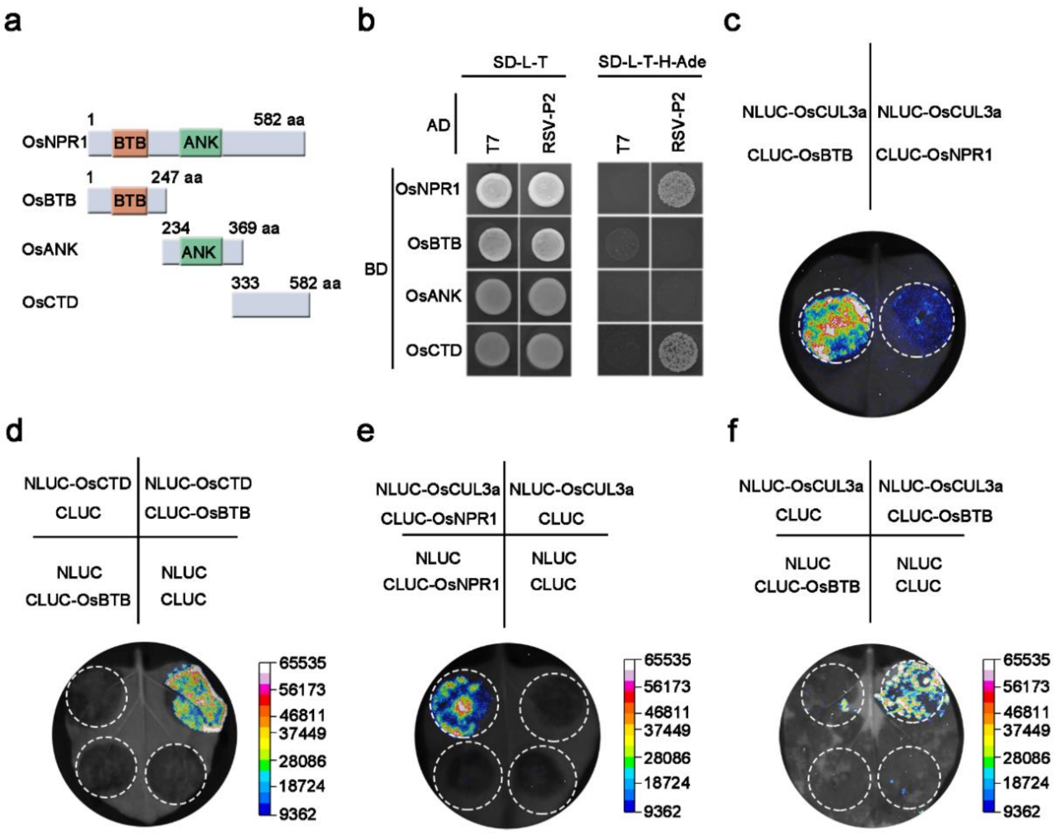

Supplementary Fig. 5. The OsNPR1 mutants for interaction with OsCUL3a and P2. a. Schematic diagrams of OsNPR1 and its deletion mutants used to test interactions with OsCUL3a or P2 protein. **b.** Y2H assay indicating the interaction between OsNPR1 variants and P2 protein. OsNPR1 and its variants were fused with BD, while RSV P2 was fused with AD yeast vectors. The different combinations were transformed into yeast cells and grown on SD-L-T plates at 30°C for 3 days. Colony growth was scanned after 3 days of incubation in SD-L-T-H-Ade medium. **c.** (Top) Scheme for Luciferase complementation imaging (LCI) assays in leaves of *N. benthamiana*. (Bottom) LCI assays showing that the interaction between BTB domain and OsCUL3a was stronger than OsNPR1. **d.** (Top) Scheme for Luciferase complementation imaging (LCI) assays in leaves of *N. benthamiana*. (Bottom) LCI assays showing that CTD was associated with BTB. **e.** (Top) Scheme for Luciferase complementation imaging (LCI) assays in leaves of *N. benthamiana*. (Bottom) LCI assays showing that OsNPR1 was associated with OsCUL3a. **f.** (Top) Scheme for Luciferase complementation imaging (LCI) assays in leaves of *N.*

benthamiana. (Bottom) LCI assays showing that BTB was associated with OsCUL3a.

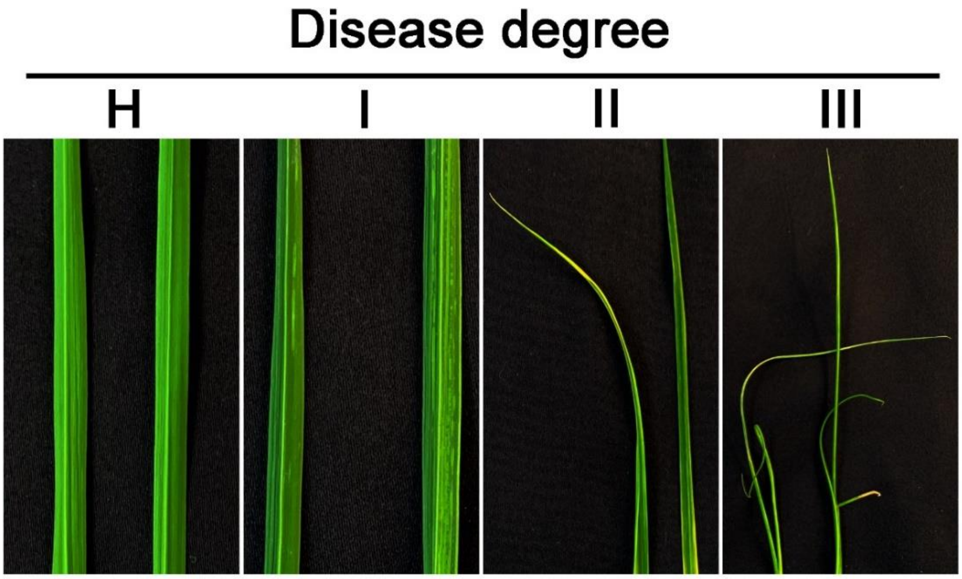

Supplementary Fig. 6. Typical disease symptoms (grade I to grade III) of RSV-infected rice plants. H: heathy plants; I: milder virus symptoms with discontinuous yellow stripes and necrotic streaks; II: typical yellow stripes and necrotic stripes; III: severe curling or death of the young leaves.

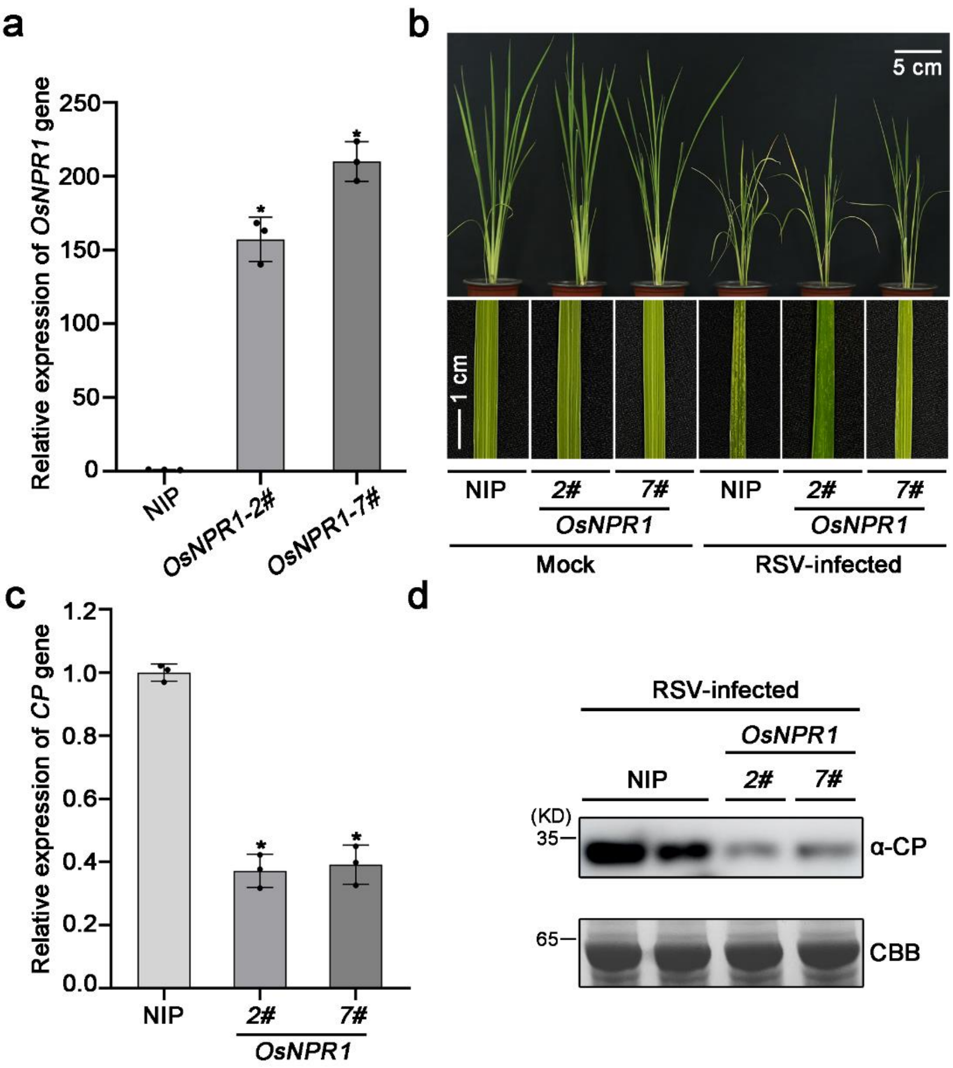

Supplementary Fig. 7. OsNPR1 enhances resistance to RSV infection in rice. a. The relative expression levels of *OsNPR1* gene in *OsNPR1* transgenic and WT (NIP) rice plants. Error bars represent SD, values are means ± SD (*n* = 3 biologically independent replicates per genotype). Significant differences were analyzed using one-way ANOVA followed by Tukey's multiple comparisons test. * at the columns indicate significant differences ($p \leq 0.05$). **b.** Viral symptoms of *OsNPR1-2#*, *OsNPR1-7#* and NIP in response to RSV infection. The phenotypes were observed and photos taken at 30 dpi. Scale bars = 5 cm or 1cm. **c.** The relative mRNA levels of RSV *CP* in RSV-infected *OsNPR1* transgenic plants and NIP rice plants as detected by RT-qPCR at 30 dpi. Error bars represent SD, values are means ± SD (*n* = 3 biologically independent replicates per genotype). Significant differences were analyzed

using one-way ANOVA followed by Tukey's multiple comparisons test. * at the columns indicate significant differences (*p* ≤ 0.05). **d.** The accumulation of RSV CP protein in RSV-infected NIP and *OsNPR1* transgenic plants determined by western blotting. CBB serves as the loading control to monitor input protein amounts. Experiments in **d** were repeated three times with the similar results. Source data including uncropped scans of gels **d** and *p* values of statistic tests (**a** and **c**) are provided in the Source data file.

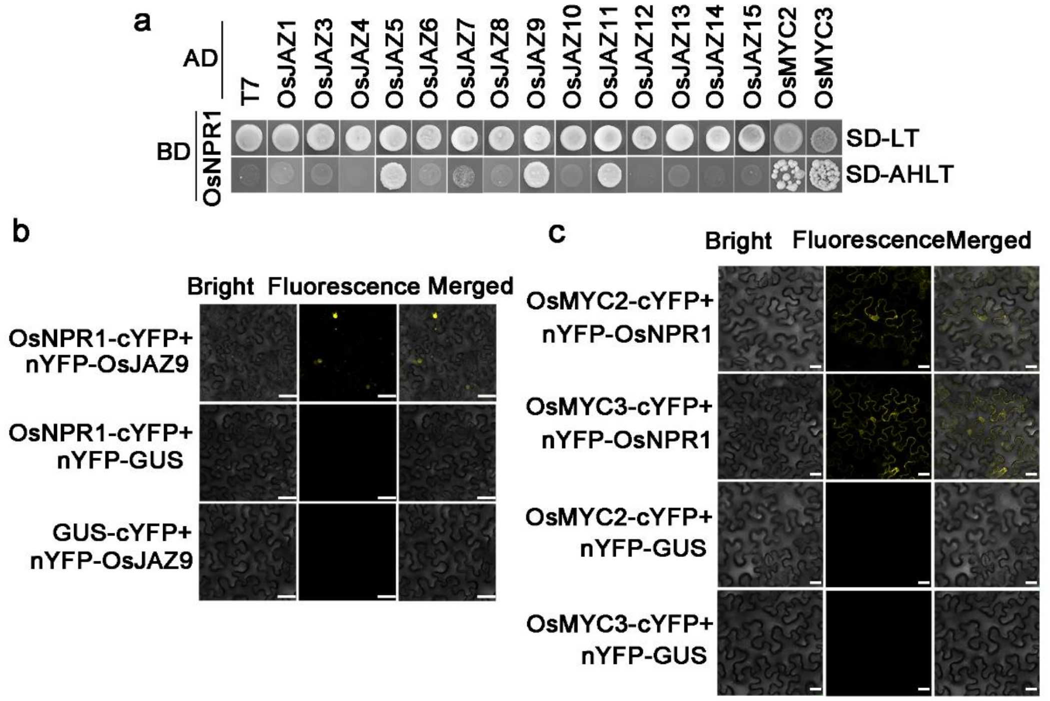

Supplementary Fig. 8. Interactions of OsNPR1 with OsJAZs and OsMYCs proteins. a. OsNPR1 interactions with OsJAZs and OsMYC2/3 proteins by Y2H assay. The 15 OsJAZ proteins except OsJAZ2 (OsJAZ1, OsJAZ3-15), OsMYC2 and OsMYC3 were cloned and tested for any interaction with OsNPR1. OsNPR1 protein was fused with BD while OsJAZs and OsMYC2/3 were fused with AD yeast vectors. The different combinations were transformed into yeast cells and grown on SD-L-T plates at 30°C for 3 days. Colony growth was scanned after 3 days of incubation in SD-L-T-H-Ade medium. The results showed that OsJAZ5, OsJAZ9, OsJAZ11, OsMYC2 and OsMYC3 interacted with P2. **b, c.** Interaction of OsNPR1 with OsJAZ9, OsMYC2 and OsMYC3 in the BiFC assays. nYFP-OsNPR1 were agro-injected together with OsJAZ9-cYFP, OsMYC2-cYFP, OsMYC3-cYFP or GUS-cYFP into *N. benthamiana* leaves, and the samples were imaged by confocal microscopy at 48 hpi. Scale bar = 50 µm. Experiments in **b** and **c** were repeated three times with the similar results.

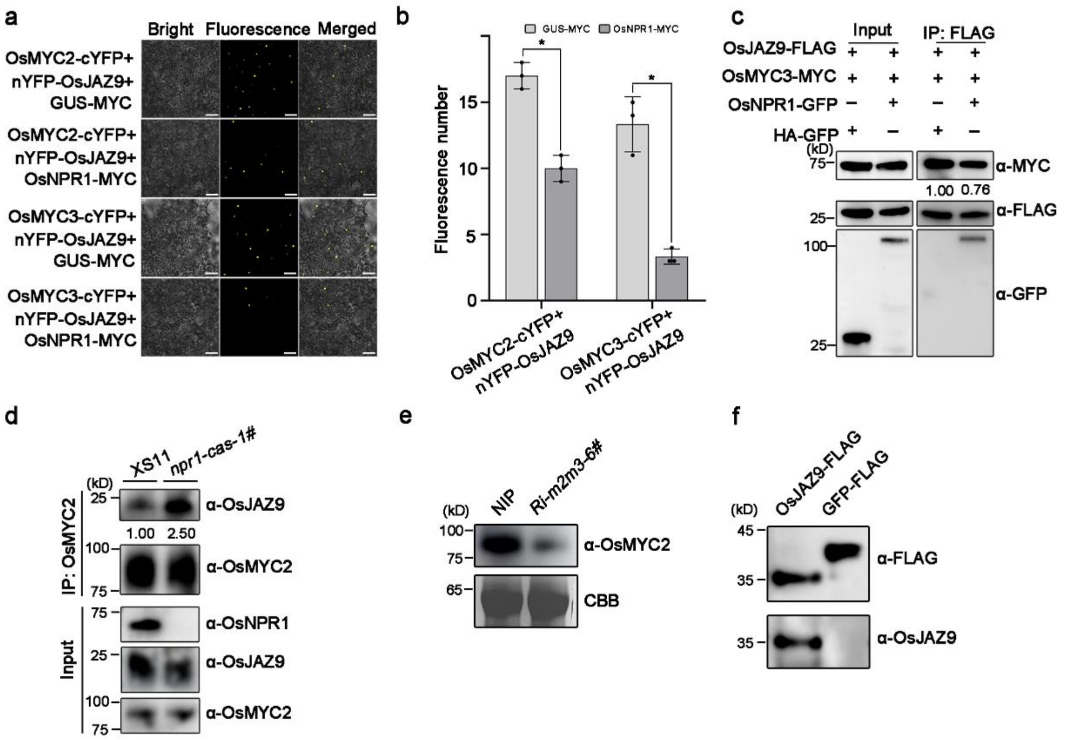

Supplementary Fig. 9. OsNPR1 disturbs the OsJAZ9-OsMYC2/3 interaction. a. OsNPR1 disturbs the association of OsMYC2/3 and OsJAZ9. The leaves were injected with 50 μM MG132, and DMSO as control at 24 hpi. The images were captured by confocal microscopy at 48 hpi. The fusion proteins were transiently expressed in leaves of *N. benthamiana* and observed by confocal microscopy at 48 hpi. Scale bar = 50 μm. **b.** The YFP signals were reduced in the presence of OsNPR1 protein. Numbers of fluorescent spots were quantified relative to the control. Error bars represent SD, values are means ± SD (n = 3 biologically independent replicates per genotype). Significant differences were analyzed using one-way ANOVA followed by Tukey's multiple comparisons test. * at the columns indicate significant differences ($p \leq 0.05$). **c.** Protein competition analyzed by Co-IP assays in *N. benthamiana*. OsJAZ9-FLAG and OsMYC3-MYC were infiltrated with or without OsNPR1-GFP into leaves of *N. benthamiana*, HA-GFP served as negative control. The samples were harvested at 48 hpi for coimmunoprecipitation with FLAG beads. **d.** Protein competition analyzed by Co-IP assays in XS11 and *npr1-cas* mutant rice plants. Total proteins were extracted, the supernatant precipitated with Protein A/G OsMYC2 antibody

beads and the immunoprecipitated (IP) and input proteins were then analyzed using anti-OsNPR1, anti-OsMYC2 and anti-OsJAZ9 antibodies. **e.** The specificity of OsMYC2 antibody using western blotting assays in NIP and *Ri-m2m3-6#* plants. Total proteins were extracted and then immunoblotted by gel blot with anti-OsMYC2 antibody. **f.** The specificity of OsJAZ9 antibody using western blotting assays in *N. benthamiana* leaves. OsJAZ9-FLAG or GFP-FLAG was expressed in *N. benthamiana* by agroinfiltration, respectively, and then the extracts were obtained for western blotting at 48 hpi, with anti-FLAG and anti-OsJAZ9 antibodies. Experiments in **a**, **c-f** were repeated three times with the similar results. Source data including uncropped scans of gels (**c-f**) and *p* values of statistic tests in **b** are provided in the Source data file.

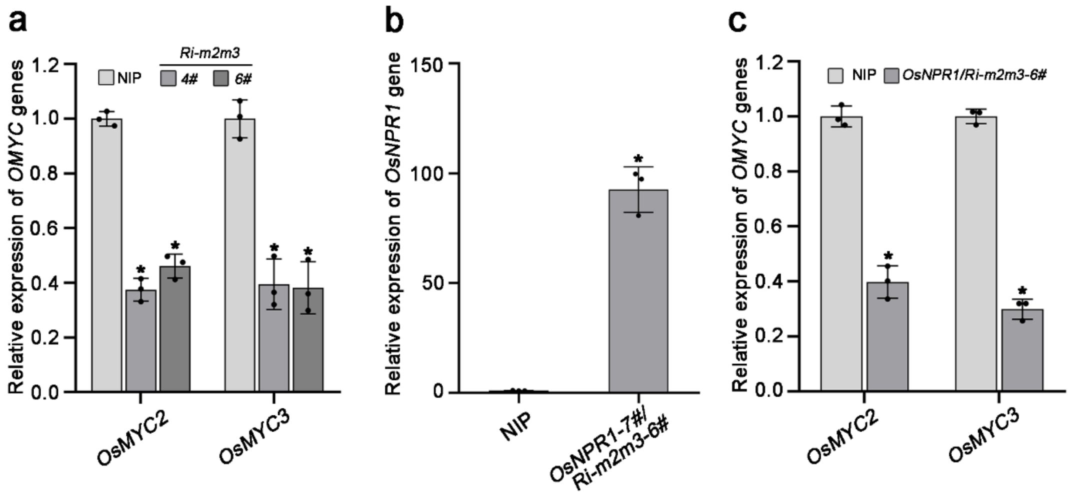

Supplementary Fig. 10. The relative expression levels of *OsNPR1*, *OsMYC2* and *OsMYC3* genes in transgenic plants. a, c. The relative expression levels of *OsMYC2* and *OsMYC3* genes in *Ri-m2m3* and *OsNPR1/Ri-m2m3* transgenic and NIP rice plants. Error bars represent SD, values are means ± SD (n = 3 biologically independent replicates per genotype). Significant differences were analyzed using one-way ANOVA followed by Tukey's multiple comparisons test. * at the columns indicate significant differences ($p \leq 0.05$). **b.** The relative expression levels of *OsNPR1* gene in *OsNPR1/Ri-m2m3* transgenic and NIP rice plants. Error bars represent SD, values are means ± SD (n = 3 biologically independent replicates per genotype). Significant differences were analyzed using one-way ANOVA followed by Tukey's multiple comparisons test. * at the columns indicate significant differences ($p \leq 0.05$). Source data including p values of statistic tests (**a-c**) are provided in the Source data file.

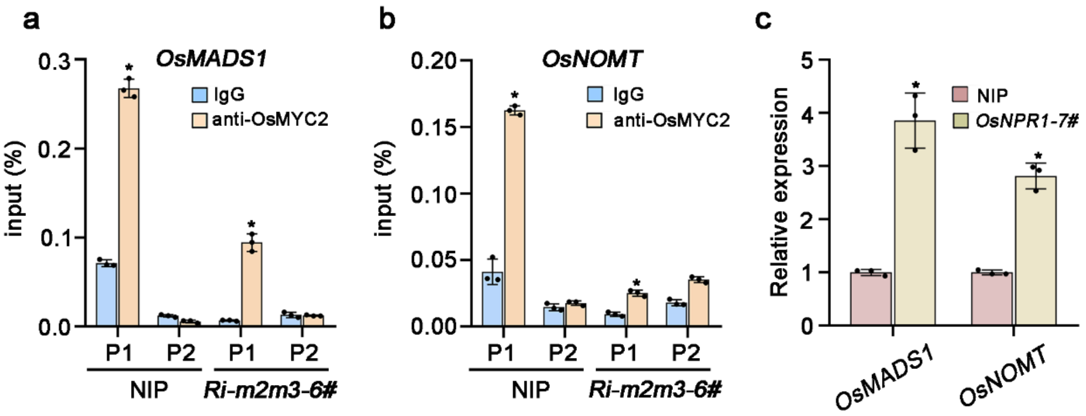

Supplementary Fig. 11. OsMYC2 specifically binds to the promoters of the *OsMADS1* and *OsNOMT* genes. a, b. ChIP-qPCR analyses of OsMYC2 binding to the G-box from *OsMADS1* and *OsNOMT* promoters in NIP and *Ri-m2m3-6#* plants using OsMYC2-specific polyclonal antibodies. Error bars represent SD, values are means ± SD (n = 3 biologically independent replicates per genotype). Significant differences were analyzed using one-way ANOVA followed by Tukey's multiple comparisons test. * at the columns indicate significant differences ($p \leq 0.05$). **c.** The relative expression levels of *OsMADS1* and *OsNOMT* genes in *OsNPR1* transgenic and NIP rice plants. Error bars represent SD, values are means ± SD (n = 3 biologically independent replicates per genotype). Significant differences were analyzed using one-way ANOVA followed by Tukey's multiple comparisons test. * at the columns indicate significant differences ($p \leq 0.05$). Source data including p values of statistic tests (**a-c**) are provided in the Source data file.

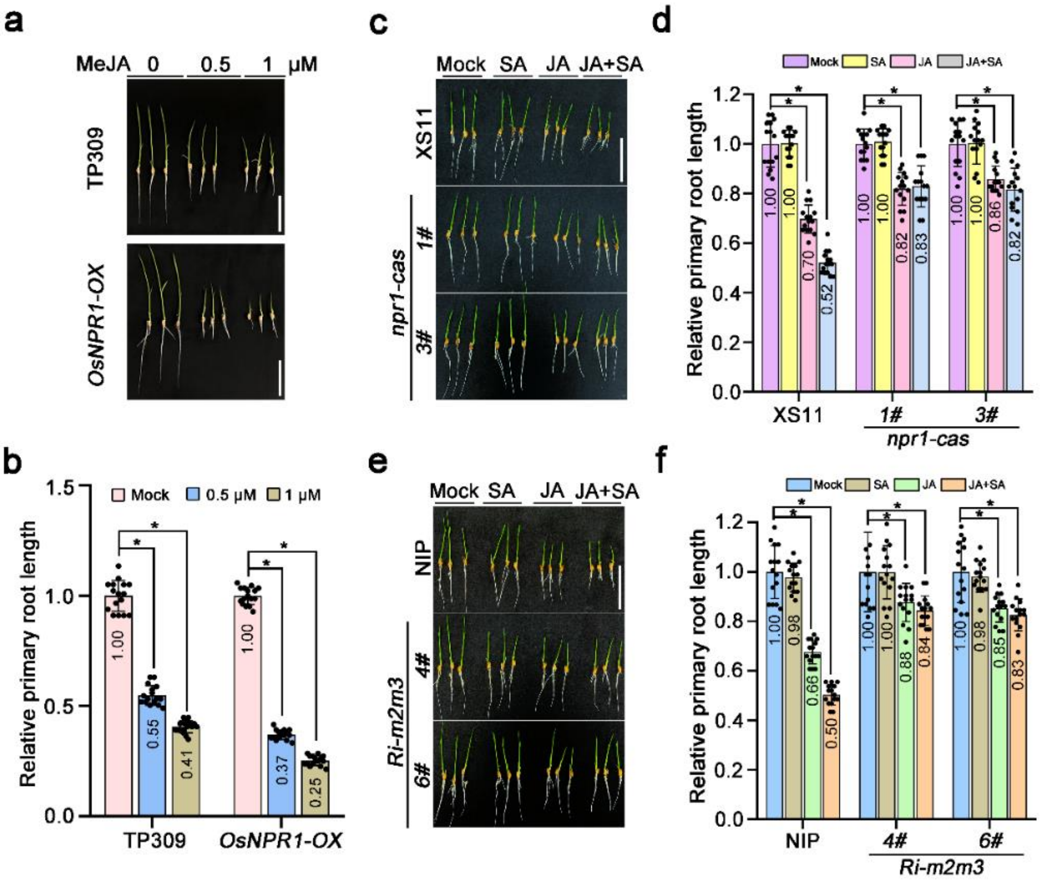

Supplementary Fig. 12. The effect of JA and SA on the primary root length of OsNPR1-related transgenic plants and *Ri-m2m3* plants. a. Phenotypes of TP309 and *OsNPR1-OX* seedlings treated with MeJA. At least 15 germinated seeds were placed in culture solution containing different concentrations of MeJA (0, 0.5 and 1 μM) for about 5 days, scale bar = 5 cm. **b.** The primary root lengths of TP309 (n = 16) and *OsNPR1-OX* (n = 16) relative to the control. Error bars represent SD, values are means ± SD. Significant differences were analyzed using one-way ANOVA followed by Tukey's multiple comparisons test. * at the columns indicate significant differences ($p \leq 0.05$). **c, e.** Phenotypes of XS11 (n = 15), *npr1-cas-1#* (n = 15) and *npr1-cas-3#* (n = 15) (c) and NIP (n = 15), *Ri-m2m3-4#* (n = 15) and *Ri-m2m3-6#* (n = 15) (e) seedlings treated with SA and/or MeJA. The germinated seeds were placed in culture solutions containing SA (1 μM) and/or MeJA (0.5 μM) for about 5 days, scale bar = 5 cm. Error bars represent SD, values are means ± SD. Significant differences were analyzed using one-way

ANOVA followed by Tukey's multiple comparisons test. * at the columns indicate significant differences ($p \leq 0.05$). **d, f.** The primary root lengths of XS11 ($n = 15$), *npr1-cas-1#* ($n = 15$) and *npr1-cas-3#* ($n = 15$) (d) and NIP ($n = 15$), *Ri-m2m3-4#* ($n = 15$) and *Ri-m2m3-6#* ($n = 15$) (f) seedlings relative to the control. Error bars represent SD, values are means ± SD. Significant differences were analyzed using one-way ANOVA followed by Tukey's multiple comparisons test. * at the columns indicate significant differences ($p \leq 0.05$). Source data including *p* values of statistic tests (**b, d** and **f**) are provided in the Source data file.

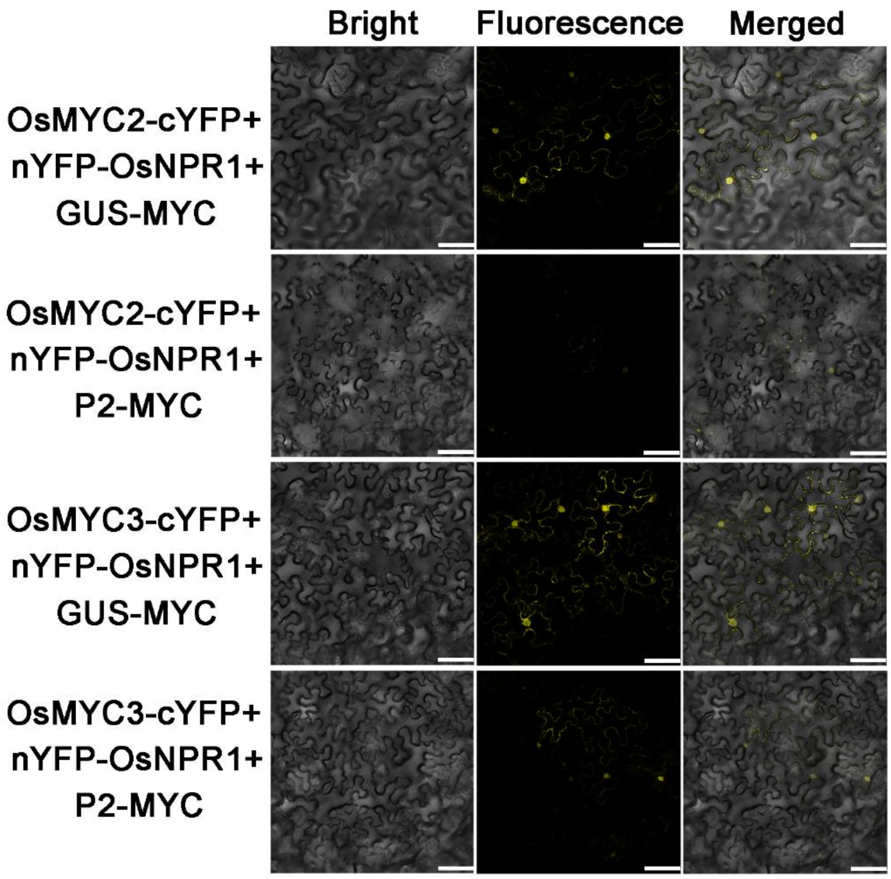

Supplementary Fig. 13. P2 disturbs the association of OsMYC2 or OsMYC3 with OsNPR1. Fusion proteins were transiently expressed in leaves of *N. benthamiana* and observed by confocal microscopy at 48 hpi. Scale bar = 50 µm. The YFP signals were reduced in the presence of P2 protein. GUS-MYC serves as the negative control. Experiments were repeated three times with the similar results.

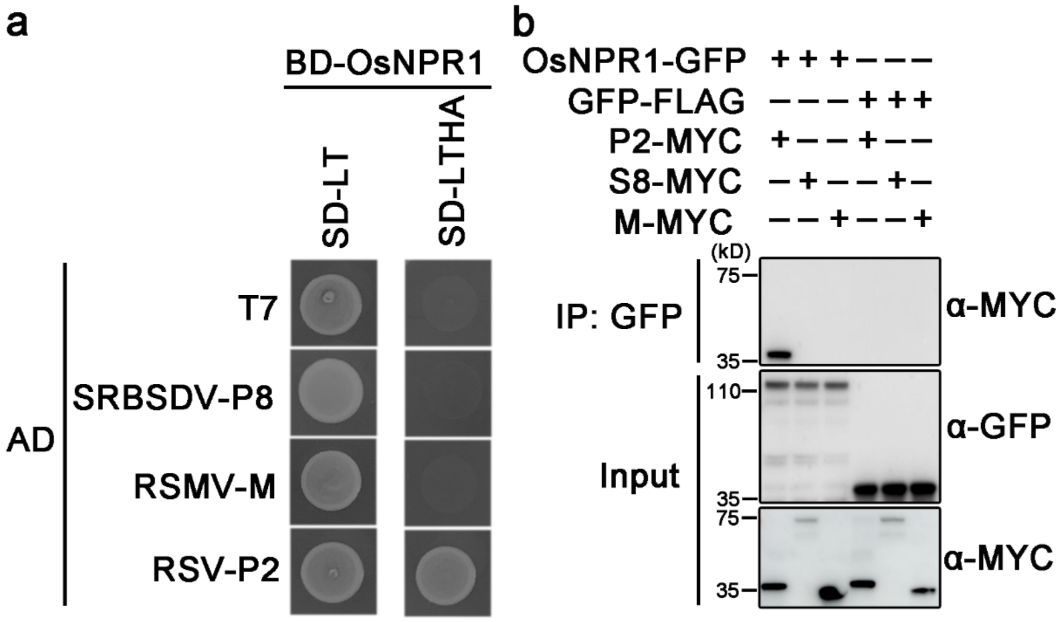

Supplementary Fig. 14. SRBSDV SP8 and RSMV M protein did not interact with OsNPR1. a. Y2H assay showing that SRBSDV SP8 and RSMV M protein did not interact with OsNPR1 in yeast cells. OsNPR1 protein was fused with BD, while SRBSDV P8, RSMV M and RSV P2 were fused with AD yeast vectors, respectively. The different combinations were transformed into yeast cells and grown on SD-L-T plates at 30°C for 3 days. **b.** Co-IP assay showing that OsNPR1 also did not associate with SRBSDV P8 or RSMV M in *N. benthamiana* leaves. Experiments in **b** were repeated three times with the similar results. Source data including uncropped scans of gels in **b** are provided in the Source data file.

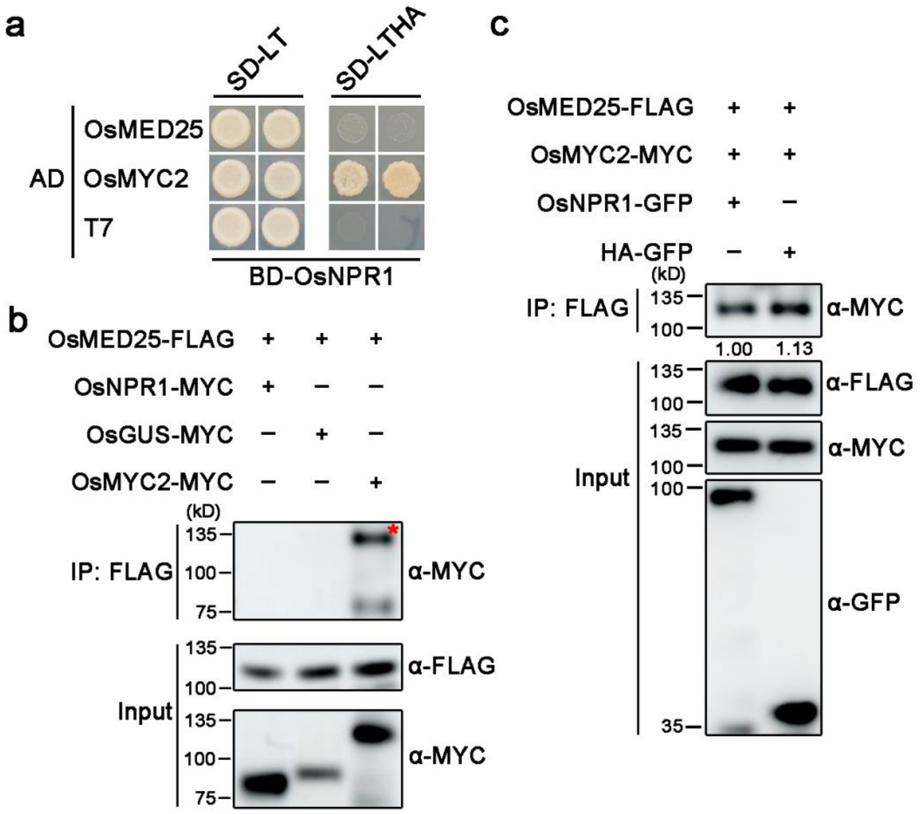

Supplementary Fig. 15. Protein competition Co-IP assays showing that OsNPR1 did not influence the OsMYC2-OsMED25 interaction. a. OsNPR1 did not specifically interact with OsMED25 in a Y2H assay. **b.** OsNPR1 did not associate with OsMED25 in Co-IP assays. Bands shown in figure are indicated by red asterisk. **c.** Protein competition Co-IP assays: OsMED25-FLAG and OsMYC2-MYC were infiltrated with or without OsNPR1-GFP into leaves of *N. benthamiana*. HA-GFP served as negative control. The samples were harvested at 48 hpi for coimmunoprecipitation with FLAG beads. Experiments in **b** and **c** were repeated three times with the similar results. Source data including uncropped scans of gels (**b** and **c**) are provided in the Source data file.

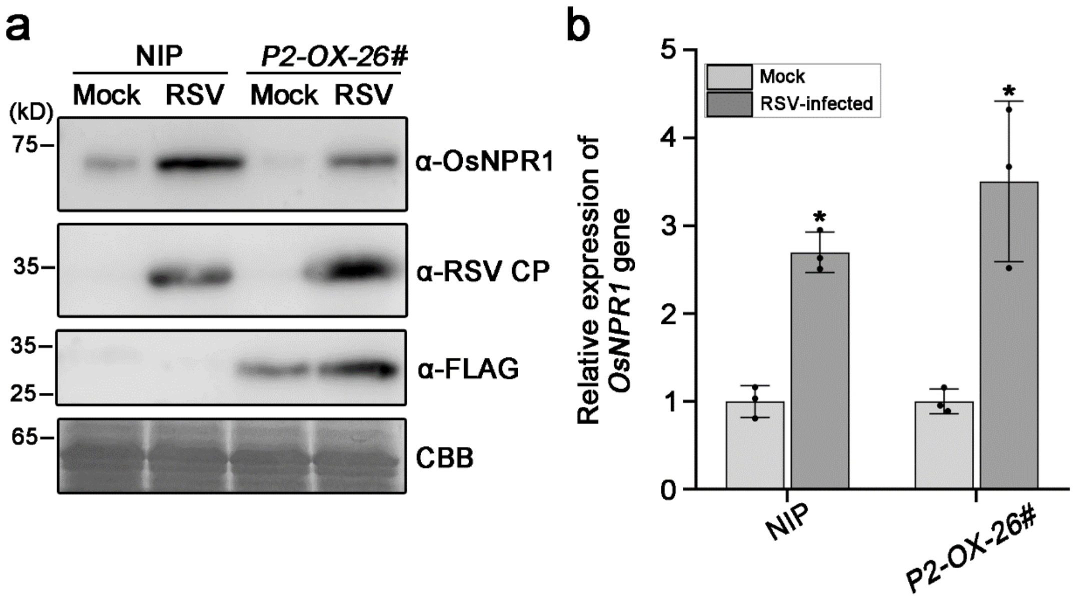

Supplementary Fig. 16. The protein and transcription levels of OsNPR1 in *P2-OX* transgenic and NIP plants after RSV infection. a. Western blotting assays showing the accumulation of OsNPR1 in RSV-infected *P2-OX* transgenic and NIP plants. **b.** Results of RT-qPCR indicating the relative expression levels of RSV *CP* in RSV-infected *P2-OX* transgenic and NIP plants. Significant differences were identified using Tukey's least significant difference tests. * at the top of columns indicates significant difference at $p \leq 0.05$. Error bars represent SD, values are means ± SD (n = 3 biologically independent replicates per genotype). Significant differences were analyzed using one-way ANOVA followed by Tukey’s multiple comparisons test. * at the columns indicate significant differences ($p \leq 0.05$). Experiments in **a** were repeated three times with the similar results. Source data including uncropped scans of gels in **a** and p values of statistic tests in **b** are provided in the Source data file.

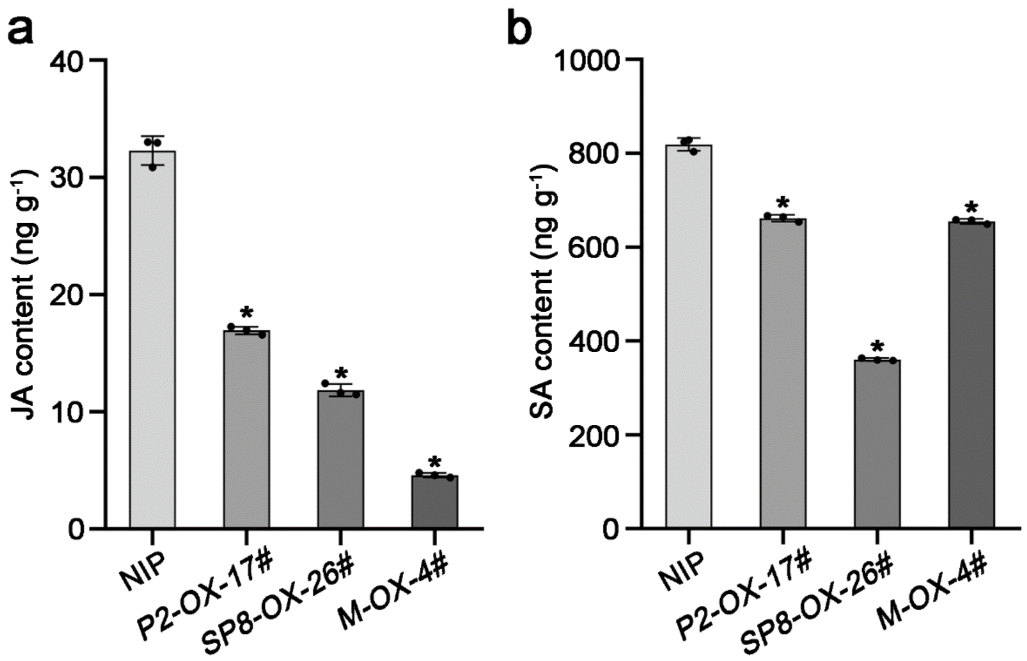

Supplementary Fig. 17. The effect of viral proteins on the JA and SA concentrations in rice leaves. a. The levels of endogenous JA content in transgenic plants expressing viral proteins (*P2-OX, SP8-OX* and *M-OX*). Error bars represent SD, values are means ± SD (*n* = 3 biologically independent replicates per genotype). Significant differences were analyzed using one-way ANOVA followed by Tukey's multiple comparisons test. * at the columns indicate significant differences ($p \leq 0.05$). **b.** The accumulation of endogenous SA content in *P2-OX, SP8-OX* and *M-OX* transgenic plants. Error bars represent SD, values are means ± SD (*n* = 3 biologically independent replicates per genotype). Significant differences were analyzed using one-way ANOVA followed by Tukey's multiple comparisons test. * at the columns indicate significant differences ($p \leq 0.05$). Source data including *p* values of statistic tests (**a** and **b**) are provided in the Source data file.

Supplementary Table 1. Primers used in this paper.

**Primers used for RT-qPCR**	
RSV CP-F	AGGCAATCAATGACATCTCC
RSV CP-R	ATCTCTCACAAAGCCAGTGC
SRBSDV S2-F	CATCGACCAAGTTCAACCCG
SRBSDV S2-R	AAGAAGTCTGCGGGTGAAGA
SRBSDV S4-F	AAAGTGAACCCGTTGCTGAC
SRBSDV S4-R	TGCAACGCTAGATCCTATGC
SRBSDV S6-F	ATCTGCTTTTCCCCTTCCGA
SRBSDV S6-R	GATTCCGCGTTTGAAGAGTCA
RSMV N-F	AGAGGTTGGAGAGGGGAAGA
RSMV N-R	TAGCCGCCCTTCTATCCTTG
OsNPR1-F	GAGCCCTTGACTCTGACGAT
OsNPR1-R	CCTCGCAGCAATGTGAAGAA
OsMYC2-F	TCGATGAACCTTTGGACGGA
OsMYC2-R	CAGCGTGTCCTGGTTGAAC
OsMYC3-F	GGCGTCCATGTACTTCTCCT
OsMYC3-R	CGGATGGCTACGACGGAA
OsMADS1-F	AGGAGCAACAGCTGCAAGAT
OsMADS1-R	GGAGAAGACCCTGATGGTGA
OsNOMT-F	CAAGCTGCTCCAATTCTTCC
OsNOMT-R	TGGGGAAGGTCGTAGTTGAC
**Primers used for ChIP-qPCR**	
chip-MADS1-P1-F	ATCTGATGTCGTGGGCAAAT
chip-MADS1-P1-R	AATGCCATCGTAAAGAGCTGA
chip-MADS1-P2-F	CGACGTGACAGCAGTTTGAT
chip-MADS1-P2-R	CTCGCTCGTCCACACACTTA
chip-NOMT-P1-F	ATGGGTCCACCGATATAAGTG
chip-NOMT-P1-R	ATCTACTAAAAGTCCATTAAACTTC
chip-NOMT-P2-F	TTGTCTCGTGATTTTCCTCCT
chip-NOMT-P2-R	GGCCTGGTTTAATTCCCTAAA
**Primers used for Y2H assays**	
BD-OsNPR1-F	ATCTCAGAGGAGGACCTGCATATGATGGAGCCGCCGACCAGCCACG
BD-OsNPR1-R	GCCGCTGCAGGTCGACGGATCCTTATCTCCTTGGTCGAATGGCCCC
BD-OsBTB-F	ATCTCAGAGGAGGACCTGCATATGATGGAACCGCCGACCAGC
BD-OsBTB-R	GCCGCTGCAGGTCGACGGATCCTTATTTAATAACATCCGGCGG
BD-OsANK-F	ATCTCAGAGGAGGACCTGCATATGATGATCACCCTGGAGAAGAG
BD-OsANK-R	GCCGCTGCAGGTCGACGGATCCTTAGGTAACATCCGCCGGA
BD-OsCTD-F	ATCTCAGAGGAGGACCTGCATATGATGTATACCGTGCTGCACAT
BD-OsCTD-R	GCCGCTGCAGGTCGACGGATCCTTATCTCCTTGGTCGAATGGCCCC
AD-OsJAZ1-F	GACGTACCAGATTACGCTCATATGGATCTGTTGGAGAAGAAG
AD-OsJAZ1-R	GCAGCTCGAGCTCGATGGATCCTTACTGGGCCTTGCCCTCAG
AD-OsJAZ3-F	GACGTACCAGATTACGCTCATATGGAGAGGGATTTTCTTGG

AD-OsJAZ3-R	GCAGCTCGAGCTCGATGGATCCTCATATCTGTAACTTTGTGCTG
AD-OsJAZ4-F	GACGTACCAGATTACGCTCATATGGAGAGGGACTTCCTGG
AD-OsJAZ4-R	GCAGCTCGAGCTCGATGGATCCTCAGATTTGTAGCTTTGTACTG
AD-OsJAZ5-F	GACGTACCAGATTACGCTCATATGTCGACGAGGGCGCC
AD-OsJAZ5-R	GCAGCTCGAGCTCGATGGATCCCTAGGACGCCGTGTGCTC
AD-OsJAZ6-F	GACGTACCAGATTACGCTCATATGGCTTCCGCGAAATCCG
AD-OsJAZ6-R	GCAGCTCGAGCTCGATGGATCCTCATTGGCTCGATTCCTGC
AD-OsJAZ7-F	GACGTACCAGATTACGCTCATATGGCGGCTTCCGCGAG
AD-OsJAZ7-R	GCAGCTCGAGCTCGATGGATCCTCATTGGCCGCGTTCTATG
AD-OsJAZ8-F	GACGTACCAGATTACGCTCATATGGCCGGCCGTGCGAC
AD-OsJAZ8-R	GCAGCTCGAGCTCGATGGATCCTCATATCTCCTGCTTTATT
AD-OsJAZ9-F	GACGTACCAGATTACGCTCATATGGCGTCGACGGATCCC
AD-OsJAZ9-R	GCAGCTCGAGCTCGATGGATCCTCAGCGCGAGTGCATGTGT
AD-OsJAZ10-F	GACGTACCAGATTACGCTCATATGGCGATGGAGGGGAAGA
AD-OsJAZ10-R	GCAGCTCGAGCTCGATGGATCCTCACAGCGCGATGGTGAG
AD-OsJAZ11-F	GACGTACCAGATTACGCTCATATGGCCGGTAGTAGCGAG
AD-OsJAZ11-R	GCAGCTCGAGCTCGATGGATCCTCACAGGCTGAGAGTGGG
AD-OsJAZ12-F	GACGTACCAGATTACGCTCATATGGCCGCCGCCGGCA
AD-OsJAZ12-R	GCAGCTCGAGCTCGATGGATCCTCAGAGCCCGAGCCATGT
AD-OsJAZ13-F	GACGTACCAGATTACGCTCATATGGCGGCGGAGGCGG
AD-OsJAZ13-R	GCAGCTCGAGCTCGATGGATCCTCAGAGCGCGAGCGCGA
AD-OsJAZ14-F	GACGTACCAGATTACGCTCATATGGCAGTGTCGGATCATCA
AD-OsJAZ14-R	GCAGCTCGAGCTCGATGGATCCTCAGTAGAACGCGGCGTC
AD-OsJAZ15-F	GACGTACCAGATTACGCTCATATGGACGCCGTCGGCGC
AD-OsJAZ15-R	GCAGCTCGAGCTCGATGGATCCTCACTTTTGCTTCCTCTTTTG
AD-OsMYC2-F	GACGTACCAGATTACGCTCATATGTGGGTTTTGTTATCTCCT
AD-OsMYC2-R	GCAGCTCGAGCTCGATGGATCCttaCCGGGCGGCGGTGC
AD-OsMYC3-F	GACGTACCAGATTACGCTCATATGTCGTGGTCCGAGACG
AD-OsMYC3-R	GCAGCTCGAGCTCGATGGATCCttaTGGAGATGGTGTAGTAAC
AD-OsMED25-F	GACGTACCAGATTACGCTCATATGGCGGCGGCGGCGGCC
AD-OsMED25-R	GCAGCTCGAGCTCGATGGATCCttaAGATAGGTAGCCACCCCCA
**Primers used for protein purification**	
6P1-NPR1-F	TCCAGGGGCCCCTGGGATCCATGGAGCCGCCGACCAGCCACG
6P1-NPR1-R	GTCAGTCACGATGCGGCCGCTTATCTCCTTGGTCGAATGGCCCC
6P1-P2-F	TCCAGGGGCCCCTGGGATCCATGATGGCATTACTCCTCTTCAAT
6P1-P2-R	GTCAGTCACGATGCGGCCGCTTAGAATAGGGCACTC
OsNPR1-MBP-HIS-F	CTGTATTTTCAGGGCCATATGATGGAGCCGCCGACCAGCCACG
OsNPR1-MBP-HIS-R	ACGGAGCTCGAATTCGGATCCTTATCTCCTTGGTCGAATGGCCCC
P2-MBP-HIS-F	CTGTATTTTCAGGGCCATATGATGGCATTACTCCTCTTCAAT
P2-MBP-HIS-R	ACGGAGCTCGAATTCGGATCCTTAGAATAGGGCACTC
OsCul3a-MBP-HIS-F	CTGTATTTTCAGGGCCATATGATGAGCGGGGGCGGGC
OsCul3a-MBP-HIS-R	ACGGAGCTCGAATTCGGATCCTGCAAGATAGCGATATAAC
**Primers used for Luciferase complementation imaging (LCI) assays**	

CLUC-OsNPR1-F	GTACGCGTCCCGGGCGGTACCATGGAGCCGCCGACCAGCCACG
CLUC-OsNPR1-R	GACGCGTACGAGATCTGGTCGACTCTCCTTGGTCGAATGGCCCC
CLUC-OsBTB-F	GTACGCGTCCCGGGCGGTACCATGGAACCGC CGACCAGC
CLUC-OsBTB-R	GAACGAAAGCTCTGCAGGTCGACTTATTTAATAACA TCCGGCGG
NLUC-OsNPR1-F	ACGAGCTCGGTACCCGGGATCCATGGAGCCGCCGACCAGCCACG
NLUC-OsNPR1-R	GACGCGTACGAGATCTGGTCGACTCTCCTTGGTCGAATGGCCCC
NLUC-OsCUL3a-F	ACGAGCTCGGTACCCGGGATCCATGAGCGGGGGCGGGC
NLUC-OsCUL3a-R	GACGCGTACGAGATCTGGTCGACTGCAAGATAGCGATATAAC
NLUC-OsCTD-F	ACGAGCTCGGTACCCGGGATCCATGTATACCGTGCTGCACAT
NLUC-OsCTD-R	GACGCGTACGAGATCTGGTCGAC ACGACGCGGA CGAATCGC
**Primers used for Dual luciferase assays**	
pGREEN-proOsMADS1-F	cttgatatcgaattcctgcagTTAAAAAAGTCAACGGCGTCAAAC
pGREEN-proOsMADS1-R	atgtttttggcgtcttccatggCCTTCTTCCTCCTCCTCTCCTCTCT
pGREEN-proOsNOMT-F	cttgatatcgaattcctgcagATGGGTCCAC CGATATAAGT
pGREEN-proOsNOMT-R	atgtttttggcgtcttccatggGGTTGTGACTTGGGTTACAA
**Primers used for BiFC and Co-IP assays**	
Lic-OsNPR1-F	CgACgACAAgACCgTCACCATGGAGCCGCCGACCAGCCACG
Lic-OsNPR1-R	gAggAgAagAgCCgTCgTCTCCTTGGTCGAATGGCCCC
Lic-OsBTB-F	CgACgACAAgACCgTCACCATGGAACCGC CGACCAGC
Lic-OsBTB-R	gAggAgAagAgCCgTCgTTTAATAACA TCCGGCGGCA
Lic-OsCTD-F	CgACgACAAgACCgTCACCATGTATACCGTGCTGCACAT
Lic-OsCTD-R	gAggAgAagAgCCgTCgACGACGCGGA CGAATCGC
Lic-△OsCTD-F	CgACgACAAgACCgTCACCATGTATACCGTGCTGCACAT
Lic-△OsCTD-R	gAggAgAagAgCCgTCgGGTAACATCCGCCGGAC
Lic-GFP-F	CgACgACAAgACCgTCACCCTGGACGGCGACGTAAAC
Lic-GFP-R	gAggAgAagAgCCgTCgGTTGTGGCGGATCTTGAAGT
Lic-P2-F	CgACgACAAgACCgTCACCATGGCATTACTCCTCTTC
Lic-P2-R	gAggAgAagAgCCgTCgCATTAGAATAGGGCACT
Lic-SP8-F	CgACgACAAgACCgTCACCATGATCGGTACATACGATGAT
Lic-SP8-R	gAggAgAagAgCCgTCgACACAGAATACTAACGGCG
Lic-M-F	CgACgACAAgACCgTCACCATGATGGCCGTTCCGTGGACT
Lic-M-R	gAggAgAagAgCCgTCgCTCCAGATTATACTTCC
Lic-GUS-F	CgACgACAAgACCgTCACCATGGTAGATC TGAGGAACCG
Lic-GUS-R	gAggAgAagAgCCgTCgGCGTTCTTGT AGCCGAAATC
Lic-OsCUL3a-F	CgACgACAAgACCgTCACCATGAGCGGGGGCGGGC
Lic-OsCUL3a-R	gAggAgAagAgCCgTCgTGCAAGATAGCGATATAACTTC
Lic-OsJAZ9-F	CgACgACAAgACCgTCACCATGGCGTCGACGGATCCC
Lic-OsJAZ9-R	gAggAgAagAgCCgTCgGCGCGAGTGCATGTGT
Lic-OsMYC2-F	CgACgACAAgACCgTCACCATGTGGGTTTTGTTATCTCCT
Lic-OsMYC2-R	gAggAgAagAgCCgTCgCCGGGCGGCGGTGCC
Lic-OsMYC3-F	CgACgACAAgACCgTCACCATGTCGTGGTCCGAGACG
Lic-OsMYC3-R	gAggAgAagAgCCgTCgTGGAGATGGTGTAGTAAC
Lic-OsMED25-F	CgACgACAAgACCgTCACCATGGCGGCGGCGGCGG

Lic-OsMED25-R	gAggAgAagAgCCgTCgAGATAGGTAGCCACCCCCA

Supplementary Table 2. Disease incidence of RSV-inoculated rice plants.

Rice plants		TP309	*OsNPR1-OX*	XS11	*npr1-cas-1#*	*npr1-cas-3#*
**III**	Repeat1	32%	23%	25%	46%	40%
	Repeat2	36%	22%	26%	52%	46%
	Repeat3	39%	23%	30%	50%	47%
**II**	Repeat1	40%	9%	40%	33%	24%
	Repeat2	36%	11%	39%	26%	29%
	Repeat3	35%	12%	30%	30%	32%
**I**	Repeat1	4%	34%	10%	13%	24%
	Repeat2	8%	30%	9%	13%	13%
	Repeat3	9%	32%	15%	10%	11%
**N**	Repeat1	24%	34%	25%	8%	12%
	Repeat2	20%	37%	26%	9%	13%
	Repeat3	17%	34%	25%	10%	11%
**Average**	III	36%	23%	27%	49%	44%
	II	37%	11%	36%	30%	28%
	I	7%	32%	11%	12%	16%
	N	20%	35%	25%	9%	12%
**SD**	III	0.03512	0.00577	0.0263	0.03222	0.03888
	II	0.02646	0.01323	0.0554	0.03627	0.03872
	I	0.02646	0.02021	0.03328	0.01623	0.07277
	N	0.03512	0.01732	0.00628	0.00877	0.01026

**Significant test objects**	**Significance test (p-value)**
III (*OsNPR1-OX* vs TP309)	3.19E-03
II (*OsNPR1-OX* vs TP309)	1.01E-04
I (*OsNPR1-OX* vs TP309)	2.07E-04
N (*OsNPR1-OX* vs TP309)	2.91E-03
III (*npr1-cas-1#* vs XS11)	4.03E-04
II (*npr1-cas-1#* vs XS11)	2.43E-01
I (*npr1-cas-1#* vs XS11)	9.86E-01
N (*npr1-cas-1#* vs XS11)	9.75E-07
III (*npr1-cas-3#* vs XS11)	1.57E-03
II (*npr1-cas-3#* vs XS11)	1.41E-01
I (*npr1-cas-3#* vs XS11)	5.19E-01
N (*npr1-cas-3#* vs XS11)	2.87E-06

Significant differences were analyzed using one-way ANOVA followed by Tukey’s multiple comparisons test.
