# Supplementary material for: Different viral effectors suppress hormone-mediated antiviral immunity of rice coordinated by OsNPR1
Source: Nat Commun. 2023 May 25;14:3011. doi: 10.1038/s41467-023-38805-x (PMC10213043; doi:10.1038/s41467-023-38805-x)
Supplement: Supplementary file 3 — Reporting Summary [file 41467_2023_38805_MOESM3_ESM.pdf]

nature portfolio

Corresponding author(s): Zongtao Sun

Last updated by author(s): May 10, 2023

Reporting Summary

Nature Portfolio wishes to improve the reproducibility of the work that we publish. This form provides structure for consistency and transparency in reporting. For further information on Nature Portfolio policies, see our Editorial Policies and the Editorial Policy Checklist.

Statistics

For all statistical analyses, confirm that the following items are present in the figure legend, table legend, main text, or Methods section.

n/a	Confirmed	
☐	☒	The exact sample size (*n*) for each experimental group/condition, given as a discrete number and unit of measurement
☐	☒	A statement on whether measurements were taken from distinct samples or whether the same sample was measured repeatedly
☐	☒	The statistical test(s) used AND whether they are one- or two-sided<br>*Only common tests should be described solely by name; describe more complex techniques in the Methods section.*
☒	☐	A description of all covariates tested
☒	☐	A description of any assumptions or corrections, such as tests of normality and adjustment for multiple comparisons
☐	☒	A full description of the statistical parameters including central tendency (e.g. means) or other basic estimates (e.g. regression coefficient) AND variation (e.g. standard deviation) or associated estimates of uncertainty (e.g. confidence intervals)
☐	☒	For null hypothesis testing, the test statistic (e.g. *F*, *t*, *r*) with confidence intervals, effect sizes, degrees of freedom and *P* value noted<br>*Give P values as exact values whenever suitable.*
☒	☐	For Bayesian analysis, information on the choice of priors and Markov chain Monte Carlo settings
☐	☐	For hierarchical and complex designs, identification of the appropriate level for tests and full reporting of outcomes
☒	☐	Estimates of effect sizes (e.g. Cohen's *d*, Pearson's *r*), indicating how they were calculated

Our web collection on statistics for biologists contains articles on many of the points above.

Software and code

Policy information about availability of computer code

Data collection	For Plant materials growth: PHCbi MLR-352H-PC;<br>For confocal microscopy: Leica TCS SP10 confocal laser microscopy;<br>For RT-qPCR: ABI7900HT Sequence Detection System (Applied Biosystems, Carlsbad, CA, USA);<br>For ChIP: Chromatin Shearing, Covaris S220;<br>For protein purification: Akta pure 25 system;<br>For protein gel Images: GE Amersham Imager 680(AI 680);<br>For firefly luciferase (LUC) complementation imaging (LCI): Low-light cooled CCD imaging apparatus (NightOWL II LB983);<br>For viral inoculation phenotype and yeast two-hybrid images: Nikon D7100 camera.

March 2021

Data analysis	The RT-qPCR , ChIP-qPCR and ultra-high-performance liquid chromatography-triple quadrupole mass spectrometry (UPLC-MS/MS) datasets were performed by Microsoft Excel 2019, Graphpad Prism 8 and Origin 8 software, the analysis using one-way ANOVA with Tukey's least significant difference tests;<br>The images from western blot and confocal microscope were cut using Adobe Photoshop CS6 and Adobe Illustrator 2020, and some information represented of their intensity were analyzed by Image J software;<br>The images from LCI were analyzed by Image J software;<br>The primary root length was manually measured by ruler and then analyzed by Graphpad Prism 8 and Origin 8 software.<br>The UPLC-MS/MS quantitative data were processed by the Masslynx V4.1 software.<br>The models were created by Adobe Photoshop CS6 and Adobe Illustrator 2020 software.

For manuscripts utilizing custom algorithms or software that are central to the research but not yet described in published literature, software must be made available to editors and reviewers. We strongly encourage code deposition in a community repository (e.g. GitHub). See the Nature Portfolio guidelines for submitting code & software for further information.

Data

Policy information about availability of data

All manuscripts must include a data availability statement. This statement should provide the following information, where applicable:

- Accession codes, unique identifiers, or web links for publicly available datasets
- A description of any restrictions on data availability
- For clinical datasets or third party data, please ensure that the statement adheres to our policy

The authors declare that all raw data supporting the findings of this study can be found within the paper and its Supplementary Files. Source data are provided with this paper.

Human research participants

Policy information about studies involving human research participants and Sex and Gender in Research.

Reporting on sex and gender	N/A
Population characteristics	N/A
Recruitment	N/A
Ethics oversight	N/A

Note that full information on the approval of the study protocol must also be provided in the manuscript.

Field-specific reporting

Please select the one below that is the best fit for your research. If you are not sure, read the appropriate sections before making your selection.

☒ Life sciences ☐ Behavioural & social sciences ☐ Ecological, evolutionary & environmental sciences

For a reference copy of the document with all sections, see nature.com/documents/nr-reporting-summary-flat.pdf

Life sciences study design

All studies must disclose on these points even when the disclosure is negative.

Sample size	For inoculation assays, each independent experiment contains 20-30 seedlings. For sensitivity assays, at least 15 transgenic seedlings were used for each line.
Data exclusions	*Describe any data exclusions. If no data were excluded from the analyses, state so OR if data were excluded, describe the exclusions and the rationale behind them, indicating whether exclusion criteria were pre-established.*
Replication	Experiment were independently repeated at least three times and similar results were obtained. The information has been provided in source data files.
Randomization	Related plant materials were grown on the same condition and randomly allocated in the growth chamber. The samples were collected randomly without any bias.
Blinding	For all experiments involving phenotypes with a randomized block design, the distribution of each family in the field was generated randomly before planting.

Reporting for specific materials, systems and methods

We require information from authors about some types of materials, experimental systems and methods used in many studies. Here, indicate whether each material, system or method listed is relevant to your study. If you are not sure if a list item applies to your research, read the appropriate section before selecting a response.

Materials & experimental systems

n/a	Involved in the study
☐	☒ Antibodies
☒	☐ Eukaryotic cell lines
☒	☐ Palaeontology and archaeology
☒	☐ Animals and other organisms
☒	☐ Clinical data
☒	☐ Dual use research of concern

Methods

n/a	Involved in the study
☒	☐ ChIP-seq
☒	☐ Flow cytometry
☒	☐ MRI-based neuroimaging

Antibodies

Antibodies used

ProteinFind® Anti-DYKDDDDK Mouse Monoclonal Antibody, TransGen, Cat#HT201-01, 1:5000 dilution;
ProteinFind® Anti-c-Myc Mouse Monoclonal Antibody, TransGen, Cat#HT101-01, 1:5000 dilution;
ProteinFind® Anti-GFP Mouse Monoclonal Antibody, TransGen, Cat#HT801-01, 1:5000 dilution;
ProteinFind® Anti-MBP Mouse Monoclonal Antibody, TransGen, Cat#HT701-01, 1:5000 dilution;
Anti-6X His tag® antibody, abcam, Cat#ab18184, 1:3000 dilution;
GST-tag Antibody [HRP], pAb, Rabbit, Genscript, Cat#A00130, 1:5000 dilution;
Ubiquitin (P4D1) mAb, Santa Cruz Biotechnology, Cat#sc-8017, 1:2000 dilution;
ProteinFind® Goat Anti-Mouse IgG (H+L), HRP Conjugate, TransGen, Cat#HS201-01, 1:10000 dilution;
ProteinFind® Goat Anti-Rabbit IgG (H+L), HRP Conjugate, TransGen, Cat#HS101-01, 1:10000 dilution;
Antin mAb for PLANTs, ab-mart, Cat#M20009, 1:5000 dilution;
Anti-Histone H3 Recombinant Rabbit Monoclonal Antibody, Huabio, Cat#ET1701-64, 1:5000 dilution.

Validation

RSV CP antibody was made from Zhejiang University;
OsNPR1, OsMYC2 and OsJAZ9 antibodies were made from our lab;
All other antibody used in this paper were certified and validated by manufactures.
The details about anti-DYKDDDK is in https://www.transgen.com.cn/antibody_tag/371.html;
The details about anti-myc is in https://www.transgen.com.cn/antibody_tag/363.html;
The details about anti-GFP is in https://www.transgen.com.cn/antibody_tag/390.html;
The details about anti-MBP is in https://www.transgen.com/antibody_tag/389.html;
The details about Anti-6X His tag® antibody [HIS.H8] is in https://www.abcam.com/6x-his-tag-antibody-hish8-ab18184.html;
The details about anti-GST is in https://www.genscript.com.cn/antibody/A00130-40-GST_tagAntibody_HRP_pAb_Rabbit.html;
The details about anti-Ubiquitin is in https://www.scbt.com/p/ubiquitin-antibody-p4d1;
The details about Goat Anti-Rabbit IgG (H+L), HRP is in https://www.transgen.com.cn/antibody_second/397.html;
The details about Goat Anti-Mouse IgG (H+L), HRP is in https://www.transgen.com.cn/antibody_second/403.html;
The details about anti-actin is in http://www.ab-mart.com.cn/list.aspx?node=59;
The details about Anti-Histone H3 antibody is in http://www.huabio.cn/product/Histone-H3-antibody-ET1701-64.
